# Molecular Dynamics Simulation of Association Processes in Aqueous Solutions of Maleate Salts of Drug-like Compounds: The Role of Counterion

**DOI:** 10.3390/ijms23116302

**Published:** 2022-06-04

**Authors:** Mikhail V. Vener, Denis E. Makhrov, Alexander P. Voronin, Daria R. Shalafan

**Affiliations:** 1 Kurnakov Institute of General and Inorganic Chemistry, Russian Academy of Sciences, Leninskii prosp. 31, 119991 Moscow, Russia; 2 Faculty of Natural Science, Mendeleev University of Chemical Technology, Miusskaya Square 9, 125047 Moscow, Russia; denis_makhrov@muctr.ru; 3 G.A. Krestov Institute of Solution Chemistry RAS, 153045 Ivanovo, Russia; apv@isc-ras.ru (A.P.V.); drs@isc-ras.ru (D.R.S.); 4 Ivanovo State University for Chemistry and Technology, 153000 Ivanovo, Russia

**Keywords:** classical MD simulations, DFT computations, ATR-IR spectroscopy, intra- and intermolecular hydrogen bonds, multicomponent organic crystals

## Abstract

The study of the formation of microstructures during the interaction of a protonated drug-like compound (API) with a maleic acid monoanion sheds light on the assembly processes in an aqueous solution at the molecular level. Molecular dynamics (MD) simulations coupled with density functional theory (DFT) calculations made it possible to find initial hydrogen bonding motifs during the assembly process, leading to the formation of heterodimers and trimers. The process of trimer formation [protonated API—maleic acid monoanion—protonated API] proceeds through the formation of three intermolecular H-bonds by the CO_2_^−^ group of the maleic acid monoanion in both systems. The total enthalpy/energy of these H-bonds is more than 70 kJ/mol. Thus, the maleic acid monoanion plays a key role in the processes of association in aqueous solution, and the interaction of the maleic acid monoanion with API is more preferable than the interaction of API molecules with each other. DFT computations in the discrete continuum approximation reveal the spectral features of heterodimers and trimers, and the ATR-IR spectra confirmed these findings. MD simulations followed by DFT calculations made it possible to describe the initial stages of the formation of pharmaceutical cocrystals in an aqueous solution.

## 1. Introduction

In the pharmaceutical industry, salt formation with a suitable counterion is considered to be one of the most preferred solutions to address the issue of low solubility of active pharmaceutical ingredients (APIs) [1,2], with hydrochlorides being a common option to consider [3,4]. Organic counterions, in particular fumaric and maleic acids [5], have gained increased attention in terms of their use in modern pharmaceutical formulations [3].

Molecular dynamics (MD) simulations with classical force fields are used to describe the dissolution process of [API + coformer] cocrystals at the atomic-molecular level [6]. However, there are practically no studies simulating the early stages of the co-crystallization process, that is, the associations of the [API + coformer] systems in aqueous solutions. The association of API molecules to form dimers or more complex supramolecular assemblies is the second step in the dissolution of pharmaceutical cocrystals [7]. The presence of coformer molecules can have an inhibitory effect on the processes of nucleation and growth of API particles due to various intermolecular interactions in solution, preventing their agglomeration and thereby reducing the rate of API crystallization and precipitation. 

**Scheme 1:**
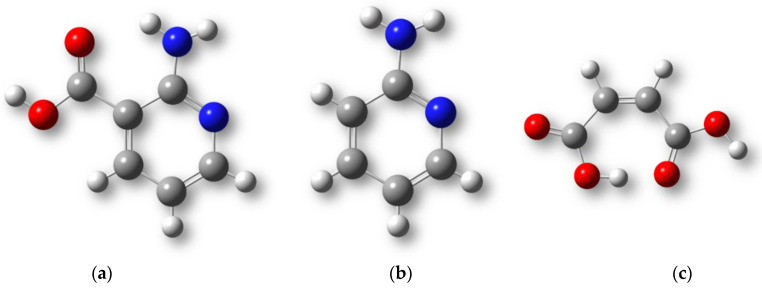
Structures of 2-aminopyridine (**a**), 2-aminonicotinic acid (**b**), and maleic acid (**c**). The red, blue, gray and white circles represent oxygen, nitrogen, carbon, and hydrogen atoms, respectively.

In this work, we studied the processes of association of the [API + coformer] and [API + coformer + H_2_O] systems in aqueous solutions by MD simulation [8]. 2-aminopyridine (2AmPyr) and 2-aminonicotinic acid (2AmNic) were chosen as model APIs, and maleic acid (Mle) as the coformer (Scheme 1). In pharmaceutical multicomponent organic crystals, maleic acid usually exists as a monoanion, while API molecules exist as cations (or protonated zwitterions) [5,9,10]. The main type of non-covalent interactions that stabilize the systems under consideration are intermolecular hydrogen bonds (H-bonds) [11,12,13,14]. When cations and anions interact in solution, short (strong) H-bonds [15] are often formed [16,17,18,19,20]. Relatively short (strong) H-bonds are formed between APIs and water molecules if the latter are part of pharmaceutical multicomponent crystals [9,19,21]. The energy of such H-bonds could be quite high (>40 kJ/mol) [19,22]. Thus, together with the primary synthons [11,23], water plays a structure-directing role in many multicomponent crystals [9,13,19,22]. It should be noted that the [2AmPyr + Mle] (1:1) crystal has nonlinear optical properties [24,25]. This salt is stabilized by N–H···O and O–H···O H-bonds apart from π–π interactions [26]. 

**Figure 1:**
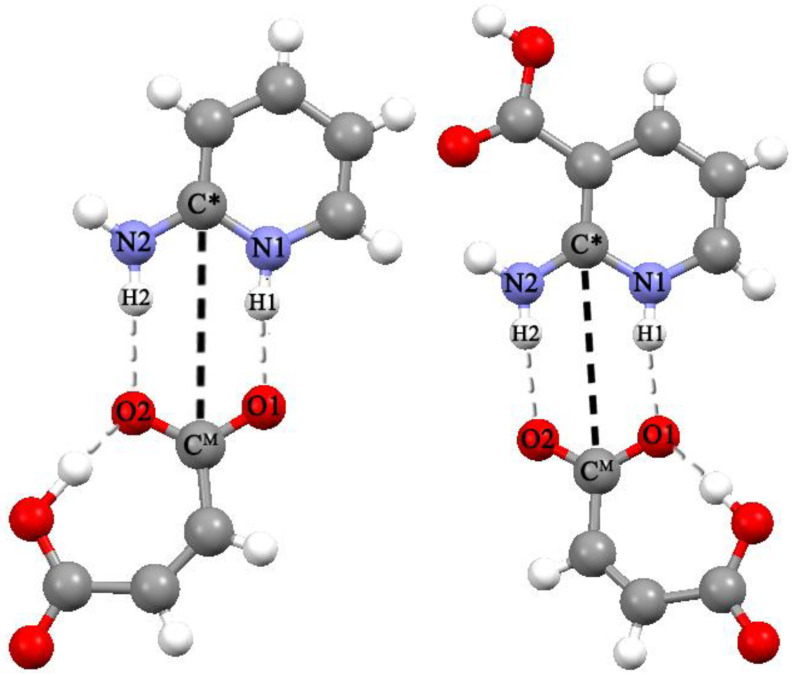
Structure of [2AmPyr + Mle] (**left**) and [2AmNic + Mle] (**right**) heterodimers isolated from [2AmPyr + Mle] (1:1) [37] and [2AmNic + Mle + H_2_O] (1:1:1) [9] multicomponent crystals. The black dotted line indicates the distance between the carbon atom of the H_2_N–C–NH group of the API cation and the carbon atom of the CO_2_^−^ group of the maleic acid monoanion, *R*[(C*···C^M^]. Gray dashed lines denote inter- and intramolecular H-bonds.

In the considered systems, a cyclic eight-membered ring with two short (strong) ^+^N–H···O^−^ bonds is realized (Figure 1). The energy of this synthon, denoted as R_2_^2^(8), is quite high (>50 kJ/mol [19]). A similar cycle is formed in the guanidinium/acetate system [27], which is a model of the so-called salt bridges [28,29]. Potentials of mean force for association of guanidinium and acetate ions were computed using different protocols for handling electrostatic forces [30] and different biomolecular force fields [31]. The existing force fields have limited applicability for the systems under consideration, since intramolecular H-bonds are realized in them. Unlike biomolecules, such H-bonds often occur in pharmaceutical cocrystals [5,32,33]. Thus, MD simulation of associations of [API + coformer] systems involves the parametrization of classical force fields for the correct description of intramolecular H-bonds of various types and strengths. 

An important issue that has to be considered is the two-step deprotonation of maleic acid. The Cambridge Structural Database version 5.42 (September 2021) [34] contains entries for three multicomponent crystals of 2-aminopyridine and maleic acid with different stoichiometric ratios of components: 2-aminopyridinium maleate (1:1 salt, refcode RONBUS), bis(2-aminopyridinium) maleate (2:1 salt, refcode WUXDEY), and 2-aminopyridinium maleate maleic acid (1:2 salt cocrystal, refcode YOVCUV). The existence of the multiple solid forms of the same API with different forms of maleic acid indicates the exceptional affinity of maleic acid towards protonated API molecules regardless of the protonation state. The variety of solid forms lies in the fact that each is obtained from solutions of different compositions [35]. Since the dissociation constants of maleic acid differ by four orders of magnitude [36], we assume that the fraction of the maleic acid dianion in the solution of [API + Mle] (1:1) is negligibly small.

This work has three aims:

(i) Develop a parametrization of classical force fields for the correct description of intramolecular H-bonds of various types and strengths. Check this on the example of aqueous solutions of two systems: [2AmPyr + Mle] and [2AmNic + Mle].
(ii) Carry out MD simulations of the early stages of the co-crystallization process, that is, the associations of the [2AmPyr + Mle] and [2AmNic + Mle] systems in aqueous solutions and describe the association process at the atomic-molecular level.
(iii) Localization of dimers, trimers, and their hydrates formed in aqueous solutions. Estimation of the average lifetime of localized structures and identification of spectral features of “long-lived” (~100 ps) associates.

## 2. Results and Discussion

In this work, both the formation of heterodimers and the formation of more complex associates (trimers) were studied. To describe the first process, it suffices to use a cubic cell containing one API cation, one maleic acid monoanion, and water. Such a cell will be called below 1 × 1 × 1. In the case of the second process, it is necessary to use a “supercell” including at least two API cations and two maleic acid monoanions. Such a supercell will be called below 2 × 1 × 1.

### 2.1. The Force Field Parameters of the Maleic Acid Monoanion

The maleic acid monoanion has a short and almost linear intramolecular O–H···O bond, the potential of which is extremely shallow [38]. The ion has a closed structure [39], and this imposes certain requirements on the parameters of the transferable force fields, which are usually not focused on the description of intramolecular H-bonds [40,41,42]. The features of the force field parameters of the maleic acid monoanion can be illustrated by comparing with those parameters of the diclofenac anion, which does not form a strong intramolecular H-bond (Figure 1 in [43]). The partial charges on the oxygen atoms of the CO_2_^−^ group of the maleic acid monoanion are –0.82 and –0.71 a.u. (The lower value corresponds to the oxygen atom forming an intramolecular H-bond). The partial charges of the oxygen atoms are equivalent and equal to –0.62 a.u. in the carboxylate group of the diclofenac anion [44].

### 2.2. The 1 × 1 × 1 Cell

**Figure 2:**
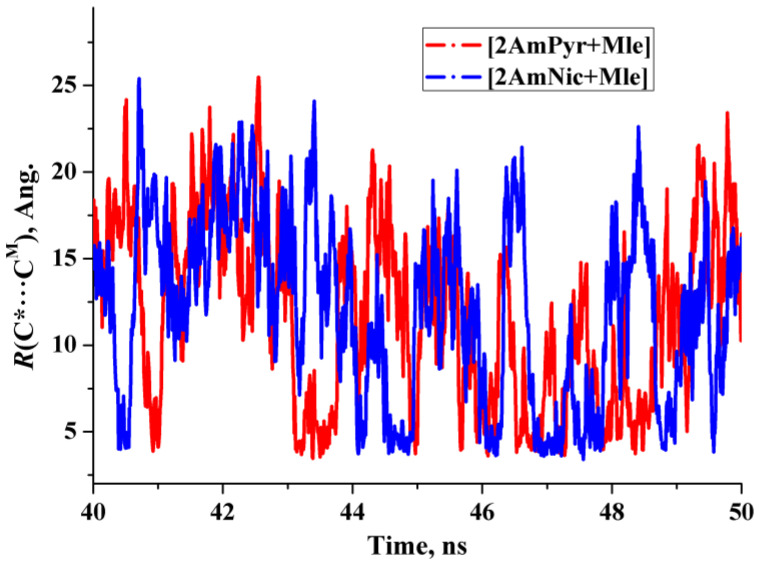
Distance R[C*···C^M^] on the part of the MD trajectories from 40 to 50 ns.

**Figure 3:**
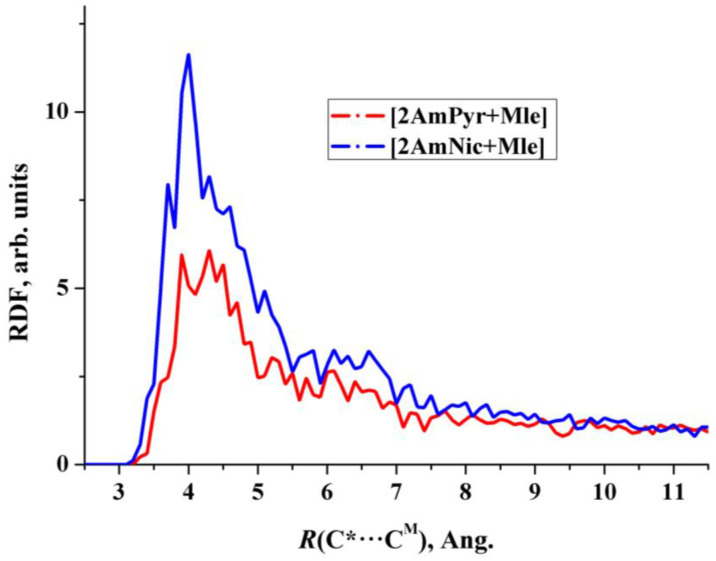
The radial distribution function (RDF) of the R[C*···C^M^] distance obtained from the 100 ns NPT simulations.

During the 100 ns NPT simulations, the API cation and the Mle anion spend a long time quite close to each other (Figure 2). In many parts of the MD trajectory, the distance C*···C^M^ is less than 5 Å for a relatively long time, ~100 ps. R(C*···C^M^) ~4.5 Å corresponds to the [2AmPyr + Mle] and [2AmNic + Mle] heterodimers (Figure 1). They are stabilized by two short ^+^N–H···O^−^ bonds (N···O distances are less than ~3 Å), forming a cyclic eight-membered ring. The radial distribution function of the R[(C*···C^M^] distance of both systems is shown in Figure 3. Both functions are characterized by a broad peak in the region of ~4–~ 4.5 Å. These values agree nicely with the R[(C*···C^M^] distances in the corresponding crystals, 4.1 Å in the [2AmPyr + Mle] (1:1) crystal [37] and 4.08 Å in crystalline [2AmNic + Mle + H_2_O] (1:1:1) [15]. Heterodimer [2AmNic + Mle] is more stable than heterodimer [2AmPyr + Mle]. (This result is a posteriori justified by our calculations, see Supplementary Materials Table S1). The potential of mean force of the considered systems is shown in Supplementary Materials Figure S1. It has a global minimum at R~4.0 Å and is associated with heterodimers with a cyclic eight-membered ring. 

To verify our results, we performed an MD simulation of the [2AmPyr + Mle] system with the TIP3P water model. The radial distance distribution function R[(C*···C^M^] obtained using the TIP3P model is compared with that obtained using the SPC/E model in Supplementary Materials Figure S2. Both functions have the same shape and peak around 4 Å.

Unlike [39], where MD simulations of ionic liquids were performed, in the present study, the Mle anion can interact with both the API cation and water molecules. The latter H-bond can be very strong for maleic acid monoanion. Obviously, the formation of stable hydrated ions will prevent the formation of heterodimers. Estimating these H-bond enthalpies is useful for describing the competition between ion hydration and dimer formation and the subsequent verification of force-field parameters of the maleic acid monoanion. The structure of the hydrated Mle anion is shown in Supplementary Materials Figure S3, and the structures of the heterodimers are shown in Figure 1. They were calculated using B3LYP/6-31G**. The polarization continuum model (PCM) was used to model the aqueous environment (Section 3.2). The enthalpies of intermolecular H-bonds were estimated using Formula (1). The total enthalpy of two ^−^O···H–O_w_ bonds formed by the C^M^O_2_^−^ group of the Mle anion with two water molecules is 43.9 kJ/mol. The total enthalpies of two ^+^N–H···O^−^ bonds formed by the Mle anion with 2AmPyr and 2AmNic cations are 48.7 and 51.2 kJ/mol, respectively, (Supplementary Materials Table S1). According to the MD simulating data, the formation of heterodimers is more favorable than the hydration of the anion, and the [2AmNic + Mle] heterodimer is more stable than the [2AmPyr + Mle] heterodimer. 

The results obtained allow us to draw the following conclusions.

(i) The force field parameters have been found to satisfactorily describe the strong intramolecular H-bond in the maleic acid monoanion, as well as the formation of heterodimers [API + Mle] in an aqueous solution;
(ii) Heterodimers [2AmPyr + Mle] and [2AmNic + Mle] exist for about 100 ps and can be treated as the long-lived species for which the IR spectrum can be evaluated [27].

### 2.3. The 2 × 1 × 1 Cell

Further association processes involve the interaction of the heterodimer with the API cation or the maleic acid monoanion. The study of H-bond networks in crystalline [2AmPyr + Mle] (1:1) and [2AmNic + Mle + H_2_O] (1:1:1) made it possible to reveal their characteristic structural motifs (Supplementary Materials Figure S4). In both crystals, trimers consisting of two API cations and a maleic acid monoanion can be isolated. One of the CO_2_^−^ groups of the maleic acid monoanion forms four H-bonds, three intermolecular and one intramolecular. An essential difference in the structure of the crystals under consideration is the presence of a water molecule in crystalline [2AmNic + Mle + H_2_O] (1:1:1). It follows from the data in Table 1 of [22] that the lone electron pair of a water molecule forms the strongest intermolecular H-bond in this crystal. (Results of the present study support this conclusion, see Supplementary Materials Table S2). The structure directional role of water was revealed in the pharmaceutical multicomponent crystals [9,22] and organic multicomponent crystals [19,45,46]. For simplicity, atoms of the second cation (cation 2) in the trimers will be denoted below by the letter “A”.

**Figure 4:**
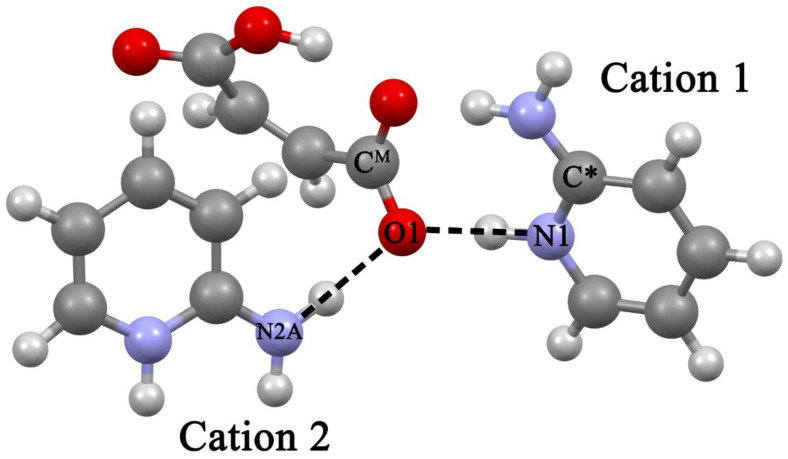
A fragment of the [2AmPyr + Mle] (1:1) crystal structure. Black dotted lines indicate the O1···N1 and O1···N2A distances.

**Figure 5:**
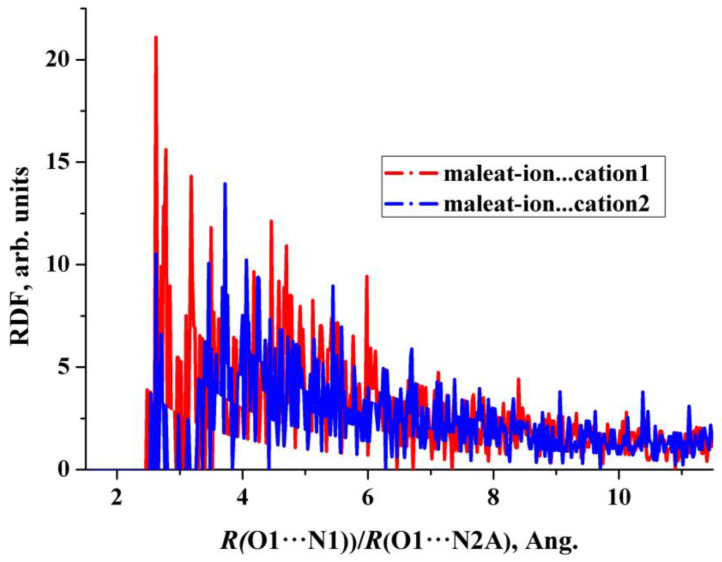
The radial distribution function (RDF) of the R[O1···N1] (red) and R[O1···N2A] (blue) distances obtained from the 100 ns NPT simulations.

In crystalline [2AmPyr + Mle] (1:1) the maleic acid monoanion forms an O1···H3A-N2A bond with energy/enthalpy greater than 20 kJ/mol (Supplementary Materials Table S2). The distance O1···N2A together with the distance O1···N1 (Figure 4) was used in MD simulation to identify trimer formation. The radial distribution functions of the O1···N1 and O1···N2A distances is shown in Figure 5. 

A maximum of about 2.7 Å on the red curve in Figure 5 corresponds to the formation of the [2AmPyr + Mle] heterodimer, while the maximum of about 3.7 Å on the blue curve corresponds to the formation of the O1···H–N2A hydrogen bond. Thus, the MD simulations indicate the formation of the [2AmPyr + Mle + 2AmPyr] trimer with the mutual arrangement of the cation 2 and the heterodimer similar to the spatial arrangement of molecules in the [2AmPyr + Mle] (1:1) crystal. Comparison of Figure 5 and Supplementary Materials Figure S5 shows that the interaction of the maleic acid monoanion with 2AmPyr is more preferable than the interaction of 2AmPyr with each other.

**Figure 6:**
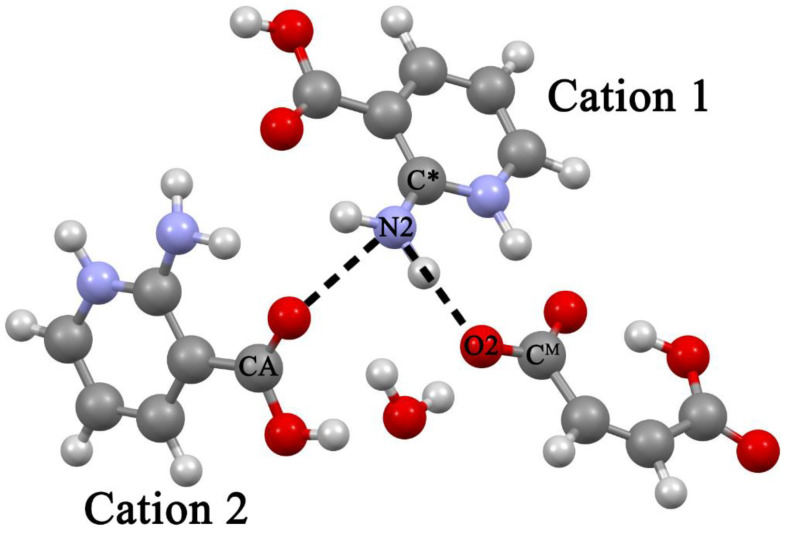
A fragment of the [2AmNic + Mle + H_2_O] (1:1:1) crystal structure. Black dotted lines indicate the N2···O2 and N2···O=CA distances.

**Figure 7:**
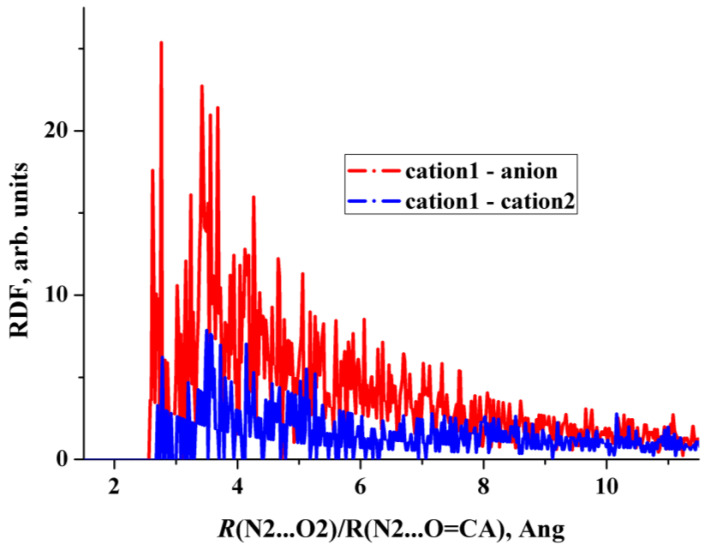
The radial distribution function of the R[N2···O2] (red) and R[N2···O=CA] (blue) distances obtained from the 100 ns NPT simulations.

The distances N2···O1 and N2···O=CA (Figure 6) were used in MD simulation to identify the formation of different associates in the case of the 2AmNic + Mle system. The radilal distribution functions of the N2···O2 and N2···O=CA distances is shown in Figure 7. A maximum of about 2.7 Å on the red curve corresponds to the formation of the [2AmNic + Mle] heterodimer, while the maximum of about 3.5 Å on the blue curve corresponds to the formation of the [2AmNic + 2AmNic] dimer. The value on the red curve at 3.5 Å is much larger than the corresponding value on the blue curve. Thus, the MD simulations indicate that the process of interaction of 2AmNic with Mle is more preferable than 2AmNic with each other. Figure 7 also indicates the formation of the [2AmNic + Mle + 2AmNic] trimer with the mutual arrangement of the cation 2 and the [2AmNic + Mle] heterodimer similar to the spatial arrangement of molecules in the [2AmNic + Mle + H_2_O] (1:1:1) crystal. The nature of the interaction of the second cation with the heterodimer is different for the systems under consideration. In the case of the 2AmPyr + Mle system, this is an intermolecular H-bond of medium strength [15] (Supplementary Materials Table S2). In the case of the 2AmNic + Mle system, the second cation interacts with the [2AmNic + Mle] heterodimer with the direct participation of a water molecule, which forms relatively strong H-bonds with the OH group of the 2 cation and the oxygen atom of the CO_2_^−^ group (Supplementary Materials Figure S4 and Table S2). A Bader electron density analysis followed by Formula (2) identified several weak intermolecular interactions between the two cations (Supplementary Materials Figure S6). The total energy of these interactions is much less than the H-bond energy between the OH group of cation 2 and the water molecule Supplementary Materials (c.f. Figure S6 with Table S2).

It should be noted that explicit consideration of the water molecule in the [2AmNic + Mle + 2AmNic] trimer is an ad hoc assumption, justified by the fact that due to the rapid exchange of water molecules, the lifetime of the associate with a certain water molecule is not large, several ps. 

### 2.4. Structural Features of Heterodimers and Trimers

This study is devoted to MD simulations of the early stages of the co-crystallization process, that is, the associations of the [API + coformer] systems in aqueous solutions. Table 1 compares the N···O/O···O distances of intermolecular H-bonds in crystal fragments with the corresponding distances in heterodimers and trimers, computed using B3LYP/6-31G** in the PCM model (water). From the data presented in the table, it follows:

(i) The calculated values of N···O distances in heterodimers differ significantly from the corresponding values in crystals. The experimental distances in the [2AmPyr + Mle] system differ by ~0.18 Å, while the calculated values differ by ~0.06 Å. The reverse pattern is observed for the [2AmNic + Mle] system (Table 1).
(ii) The explicit allowance for the second cation in trimers leads to the fact that the calculated N···O distances of the cyclic eight-membered H-bonded ring of both systems agree with experiment much better than the corresponding values in heterodimers.

An essential feature of trimers is that the oxygen atoms of the CO_2_^−^ group of the maleic acid monoanion form four H-bonds, three of which are intermolecular. The total enthalpy/energy of intermolecular H-bonds is more than 70 kJ/mol (Supplementary Materials Table S2). That is, the association process proceeds through the formation of three intermolecular H-bonds by the CO_2_^−^ group of the maleic acid monoanion in both systems. The results obtained indicate a key role of the maleic acid monoanion in association processes in aqueous solution, since the formation of its heterodimers with APIs is more preferable than the formation of API dimers.

The processes of further aggregation, that is, the formation of nanoscale structures, require the use of other theoretical approaches, for example, nucleation theory [47]. The use of such approaches is beyond the scope of this work.

### 2.5. ATR IR Spectra of Aqueous Solutions of [2AmPyr + Mle]

According to the performed calculations in the PCM model, the heterodimers and trimers of the considered systems are characterized by an intense IR band in the region of 2800 cm^−1^ due to the stretching vibrations of the N–H group of cyclic eight-membered H-bonded ring (Tables S1 and S2). An intense IR band at about 2500 cm^−1^ exists in the considered systems. This is due to vibrations of the intramolecular H-bond of the maleic acid monoanion. Below, only the [2AmPyr + Mle] dimer will be considered, since it is a “long-lived” particle for which the IR spectrum can be measured.

The transition from the PCM model to the discrete-continuum approximation (Supplementary Materials Figure S7) has little effect on the value of the wave numbers and the IR intensity of the bands caused by vibrations of the groups forming the cyclic eight-membered H-bonded ring. The IR active band due to intramolecular H-bond vibrations in the maleic acid monoanion lies at about 2150 cm^−1^ in the discrete-continuum approximation. Thus, it falls into the range of 2400 to 1900 cm^−1^, which includes the bands due to vibrations of the CO_2_ molecule (Section 3.4).

**Figure 8:**
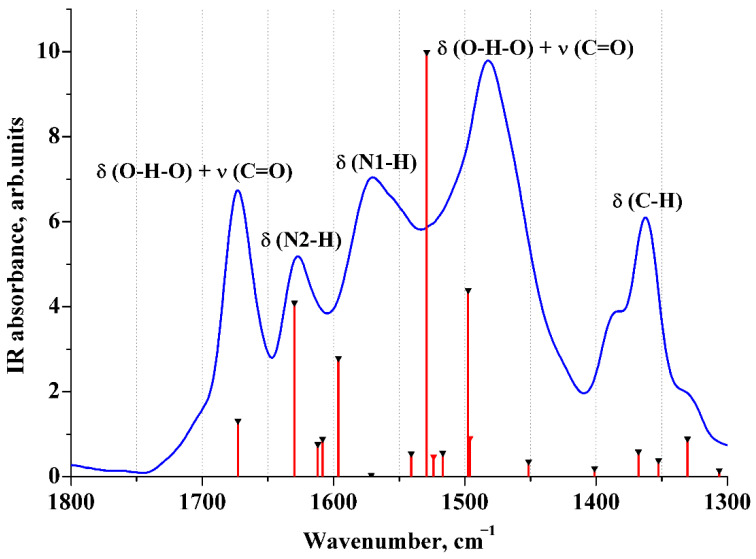
Experimental ATR IR spectra of aqueous solution of [2AmPyr + Mle] in the range of 1800–1300 cm^−1^. The calculated vibrations are given by sticks. Their height is proportional to the theoretical IR intensity. δ denotes in-plane bending vibrations, ν–stretching vibrations.

Experimental ATR IR spectrum of aqueous solution of [2AmPyr + Mle] in the range of 1800–1300 cm^−1^ is compared with theoretical spectrum, evaluated in the discrete-continuum approximation, in Figure 8. The agreement is reasonable. The most intense band in this range is due to vibrations of the intramolecular H-bond of the maleic acid monoanion strongly coupled with the asymmetric stretching vibrations of the CO_2_^−^ group.

**Figure 9:**
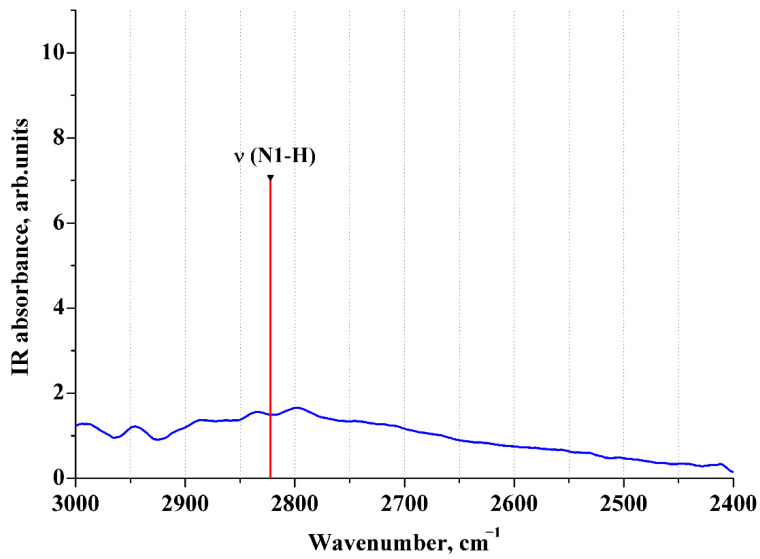
Experimental ATR IR spectra of aqueous solution of [2AmPyr + Mle] in the range of 3000–2400 cm^−1^. The computed N1–H stretching vibration is given by a stick. Its height is proportional to the theoretical IR intensity.

The N1–H group forms relatively short (strong) H-bond in the considered dimer and locates the IR active band of such bonds, often characterized by broad continuous absorption [48,49]. DFT calculations of IR intensities are usually performed in the “double harmonic approximation” [50]. It has limited applicability for estimating the frequencies and IR intensities of stretching vibrations of OH/NH groups involved in the formation of short (strong) H-bonds due to significant mechanical [51,52,53,54] and electro-optical anharmonicity [55]. A theoretical description of the considered bands beyond the “double harmonic approximation” in condensed phase (molecular crystals) is a time-consuming procedure [56,57,58]. Due to technical difficulties, such calculations in aqueous solutions are carried out for simple organic molecules [59,60]. From Figures 6 and S10 of [61], it follows that the explicit allowance for anharmonic effects leads to a sharp decrease in intensity and a strong broadening of the band due to stretching vibrations of the group forming a short (strong) H-bond. Thus, we attribute the broad band in the range of ~2950–~2600 cm^−1^ to the stretching vibrations of the N–H groups, which form the cyclic eight-membered H-bonded ring (Figure 9).

Comparison of the spectrum of [2AmPyr + Mle] with the spectrum of an aqueous solution of 2AmPyr of the same concentration is shown in Supplementary Materials (Figure S8). An intense IR band at ~2580 cm^−1^ in the aqueous solution of Mle is due to vibrations of the intramolecular H-bond of the maleic acid monoanion. In accordance with our computations, this band shifts to ~1500 cm^−1^ as a result of the formation of the heterodimer [2AmPyr + Mle].

## 3. Materials and Methods

### 3.1. Classical MD Simulations

A cubic cell (the 1 × 1 × 1 cell) is prepared containing 1 API cation, 1 maleic acid monoanion, and 1000 water molecules. This corresponds to the mass concentration of the components ~(5 − 7) × 10^−3^ g/mL. Cube edge is found as a result of simulations in the NPT ensemble. Isothermal compressibility of water equals to 4 × 10^−5^ per unit atmospheric pressure. Cube edge equals to ~31 Å. 

Atomic partial charges of the 2AmPyr, 2AmNic cations, and maleic acid monoanion are obtained using a web-based generator [62]. In this case, the data of quantum chemical calculations (B3LYP/cc-pvDz) are used. Van der Waals parameters of maleic acid monoanion are obtained using a web-based automatic parameter generator LigParGen [63]. The torsion angles for the maleate ion are taken from the quantum chemical data (B3LYP/cc-pvDz) [64]. It is assumed that the harmonic approximation adequately describes structures near local equilibrium. Topological files for 2AmPyr, 2AmNic, and Mle are given in SI. 

A 2 × 1 × 1 supercell containing 2 cations of API, 2 maleic acid monoanions and 2000 water molecules was used to study the formation of trimers and their hydrates. 

The GROMACS code [14,65,66] was used to perform the MD simulations. The force field OPLS-AA [67] was used together with the SPC/E water model [68]. To check the sensitivity of the results to force fields, the TIP3P water model [69] was also considered. The simulations were carried out in the NPT ensemble. The temperature maintained at 310 K employing the velocity-rescaling temperature coupling [70] with the time constant of 0.5 ps. The equations of motion were integrated using the leap-frog algorithm [71] with a time step of 2 fs. Long-range electrostatic interactions were calculated using the particle mesh Ewald method [72,73] (the cutoff was set at 10 Å); van der Waals and short-range interactions were truncated at 10 Å. The fluctuations of kinetic, potential, and total energy around some mean values serves as a criterion of the equilibration of the systems (100 ns, a time step is 2 fs). For the data collection, 100 ns (a time step is 2 fs) simulations were performed.

### 3.2. DFT Computations

The structure of heterodimers and trimers were computed at the B3LYP/6-31G** level using Gaussian 16 software package [74]. The PCM model was applied to simulate water medium [75]. Analysis of normal vibrations did not reveal imaginary frequencies for all calculated structures. 

IR spectra of the hydrated maleic acid monoanion, 2-aminopyridine cation, [2AmNic + Mle] dimer were computed in the discrete-continuum model [76] which is often used for the evaluation of the vibrational frequencies of molecules in water [77,78]. The number of water molecules taken into account explicitly in the calculation of the IR spectrum was equal to the number of unshared electron pairs and N-H groups that do not form H-bonds. The scaled factor was equal to 0.9648 [79].

### 3.3. Enthalpy and Energy of Intermolecular H-Bonds

Various approaches can be used to estimate the enthalpy and energy of intermolecular H-bonds, for example, see [80]. In the present work, we used approaches that explore the metric [81] and spectroscopic [82] characteristics of this bond, calculated using the B3LYP/6-31G** level in the PCM approximation (Section 3.2). 

The H-bond enthalpy (−ΔH_HB_) was estimated as previously shown [81]:

−ΔH_HB_ (kJ mol^−^^1^) = 0.134·R(O∙∙∙H)^−3.05^ (1)

where the R(H∙∙∙O) is the H∙∙∙O distance (nm).

Energy E_HB_ of intermolecular H-bonds was evaluated using the Alkorta approach [82]:

E_HB_ (kJ mol^−^^1^) = 1124·G_b_ (atomic units) (2)

where G_b_ is the positively defined local electronic kinetic energy density at the O∙∙∙H bond critical point [83]. Bader analysis of electron density was performed using AIMALL [84].

### 3.4. ATR-IR Spectroscopy

ATR-IR spectra of the system were recorded with a Bruker Vertex 80v Fourier spectrometer (Bruker Optics, Ettlingen, Germany) in the range of 4000 to 400 cm^−1^ using the MVP 2 SeriesTM (Harrick) ATR unit with a diamond crystal at room temperature with an increment of 1 cm^−1^ in the air atmosphere. The initial stock solutions of 2AmPyr, Mle, and [2AmPyr + Mle] were prepared by weighting the solid sample and bidistilled water. A row of ten solutions for ATR-IR studies for each system was prepared by dilution of a stock solution with component concentrations ranging from 0.0015 to 0.015 mole fractions in order to take into account the changes in solution density. Unfortunately, the aqueous solubility of 2AmNic and [2AmNic + Mle] was too low to record a spectrum of desired quality. The 2400 to 1900 cm^−1^ range in the ATR-IR spectra is not informative due to the uncompensated atmospheric correction for adsorbed CO_2_ molecules on the surface of the diamond crystal. 

The raw spectra were processed by subtracting the spectra of the blank water sample used for solution preparation multiplied by mole fraction of water in the system. The resulting blank-corrected spectra were normalized by concentration, and the concentration-independent average was derived from ten experiments. No visible concentration effects on band position were recorded for the bands in the range of 3000 to 2400 and 1800 to 1300 cm^−1^, indicating the stability of the complex in the whole concentration range.

## 4. Conclusions

The force field parameters have been found that satisfactorily describe the strong intramolecular H-bond in the maleic acid monoanion, as well as the formation of heterodimers [protonated API—maleic acid monoanion], where API is 2-aminopyridine or 2-aminonicotinic acid, in an aqueous solution. This is of great methodological importance, since “standard” classical force fields are oriented towards biomolecules containing practically no short (strong) intramolecular H-bonds, while these bonds are widely represented in multicomponent pharmaceutical crystals.

The process of trimer formation [protonated API—maleic acid monoanion—protonated API] proceeds through the formation of 3 intermolecular H-bonds by the CO_2_^−^ group of the maleate ion in both systems. The total enthalpy/energy of these H-bonds is more than 70 kJ/mol. Thus, the maleic acid monoanion plays a key role in the processes of association in aqueous solution, since its interaction with the API is preferable to the interaction of the API molecules with each other. 

The heterodimers of the considered systems in an aqueous solution should be characterized by a broad IR band at about 2800 cm^−1^, due to the stretching vibrations of the N–H groups of the cyclic eight-membered H-bonded ring. An intense IR band at ~ 1500 cm^−1^ is also observed, due to vibrations of the intramolecular H-bond of the maleic acid monoanion strongly coupled with the asymmetric stretching vibrations of the CO_2_^−^ group.

## Figures and Tables

**Table 1:** The N···O/O···O distances of intermolecular H-bonds in crystal fragments vs. the corresponding distances in heterodimers and trimers, computed using B3LYP/6-31G** in the PCM model (water).

Distance/Angle ^a^	X-ray	Heterodimer	Trimer
The [2AmPyr + Mle] System			
N1···O1, Å	2.727	2.823	2.781
N2···O2, Å	2.902	2.883	2.970
O1···N2A, Å	2.900	-	2.879
Dihedral angle, deg	102.1	-	88.2
The [2AmNic + Mle] System			
N1–H1···O1, Å	2.816	2.765	2.810
N2–H2···O2, Å	2.816	2.901	2.880
N2-H2···O=CA, Å	3.01	-	3.31
O2···H-Ow, Å	2.717	-	2.702
Ow···H-OA, Å	2.567	-	2.559
Dihedral angle, deg	17.4	-	43.3

^a^ The atomic numbering and dihedral angle are given in Supplementary Materials Figure S4.

## Data Availability

The IR spectra and I/O files are available from the corresponding author upon reasonable request.

## Supplementary Materials

The Supplementary Materials are available online at www.mdpi.com/article/10.3390/ijms23116302/s1. References [30,31,85,86,87,88] are cited in Supplementary Materials.
