# Supplementary material for: Molecular Dynamics Simulation of Association Processes in Aqueous Solutions of Maleate Salts of Drug-like Compounds: The Role of Counterion
Source: Int J Mol Sci. 2022 Jun 4;23(11):6302. doi: 10.3390/ijms23116302 (PMC9181654; doi:10.3390/ijms23116302)
Supplement: Supplementary file 1 [file ijms-23-06302-s001.zip › ijms-1739472-supplementary.pdf]

SI

Molecular dynamics simulation of association processes in aqueous solutions of maleate salts of drug-like compounds. The role of counterion.

Mikhail V. Vener[1]*, Denis E. Makhrov[2], Alexander P. Voronin[3], Daria R. Shalafan[3,4]

1- Kurnakov Institute of General and Inorganic Chemistry, Russian Academy of Sciences, Leninskii prosp. 31, Moscow 119991, Russia; vener@igic.ras.ru

2- Faculty of Natural Science, Mendeleev University of Chemical Technology, Miusskaya Square 9, Moscow 125047, Russia; Denis_Makhrov@muctr.ru

3- G.A. Krestov Institute of Solution Chemistry RAS, 153045, Ivanovo, Russia; apv@isc-ras.ru

4- Ivanovo State University for Chemistry and Technology, 153000, Ivanovo, Russia; drs@isc-ras.ru

Section S1.

To evaluate the relative stability of heterodimers in comparison to solvate-separated ions, the classical potential of mean force $W(R)$ was calculated using [s1]:

$$W(R) = -k_B T \ln\{\mathrm{RDF}(R)\}, \qquad \text{(S1)}$$

where k_B is the Boltzmann constant, T is the temperature, and RDF(R) is the radial distribution function obtained from the MD calculations (Fig. 3). The classical potential of mean force for both systems is shown in Figure S1. The generated classical potential of mean forces show the expected features for association of polyatomic organic ions in water [s2, s3, s4, s5, s6].

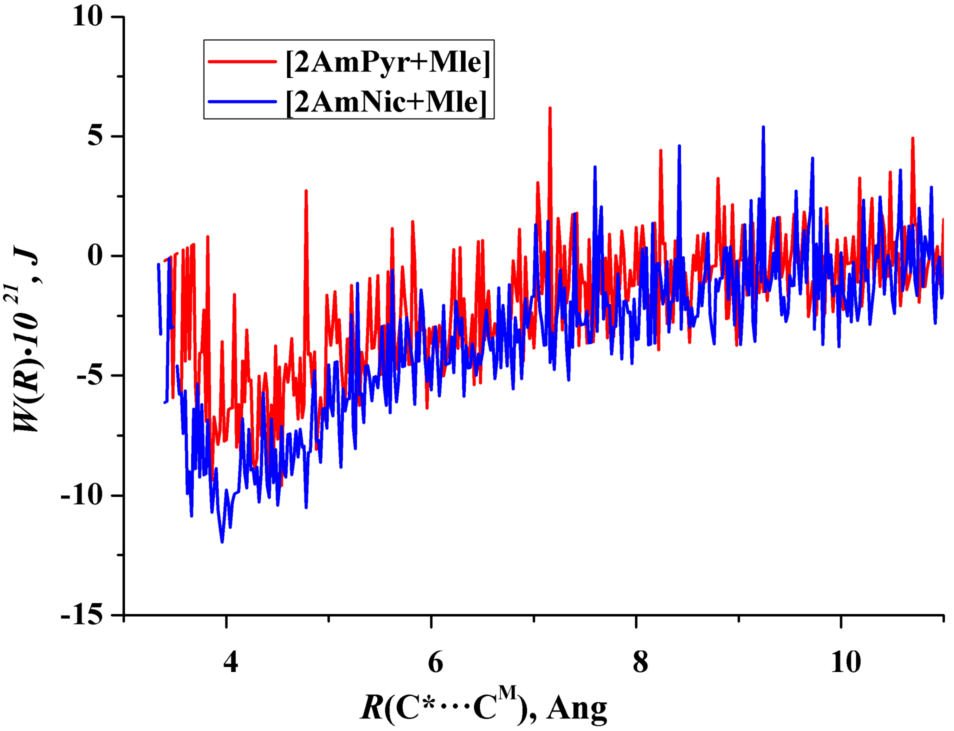

Figure S1. The classical potential of mean force *W*(*R*) for the [2AmPyr + Mle] and [2AmNic + Mle] systems.

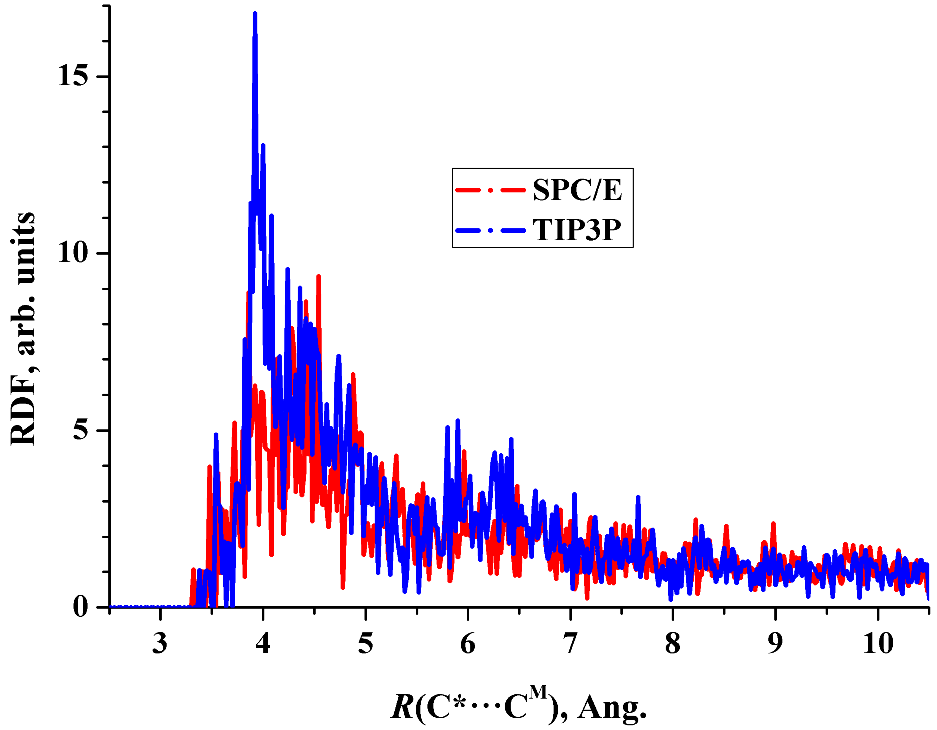

Figure S2. The radial distribution function (RDF) of the R[C*···C^{M}] distance of the [2AmPyr + Mle] system obtained from the 100 ns NPT simulations with SPC/E and TIP3P models of water.

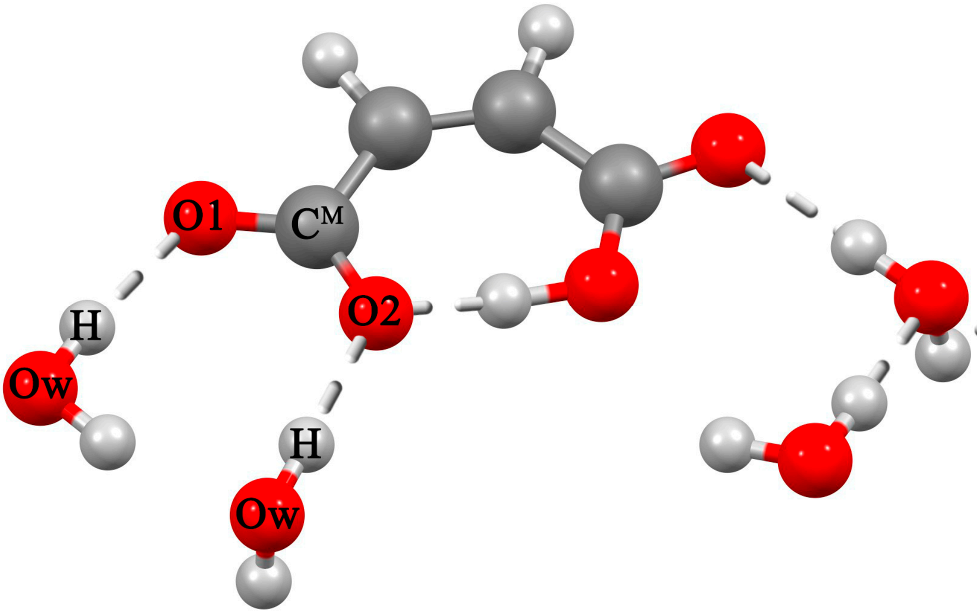

Figure S3. The structure of the hydrated Mle anion.

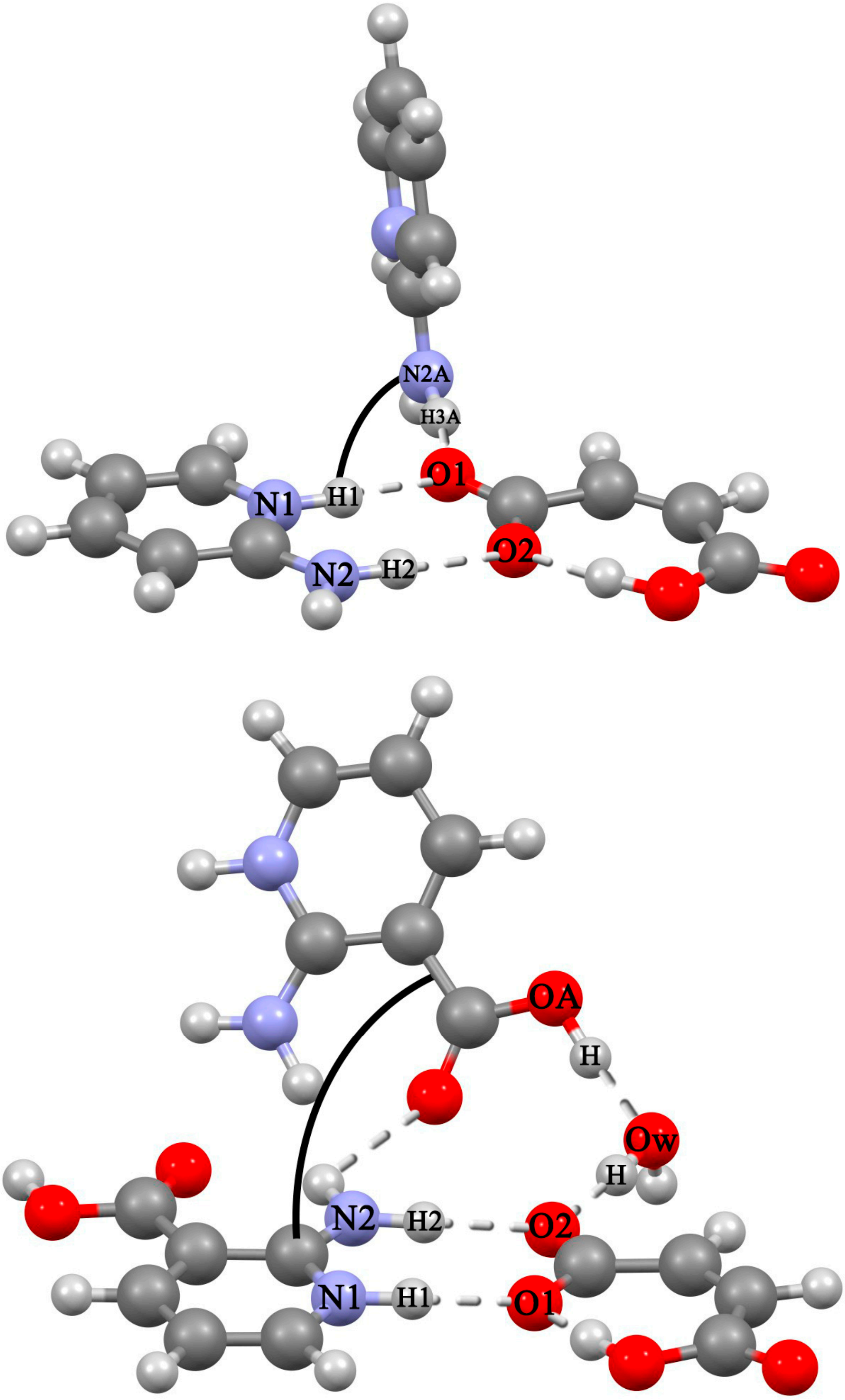

Figure S4. H-bond networks in crystalline [2AmPyr+Mle] (1:1) [37] (upper panel) and [2AmNic+Mle+H_2O] (1:1:1) [9] (lower panel). H-bonds are denoted by dotted lines.

Table S1. B3LYP/6-31G** values of the (H···O) distance R(H···O), the electron density ρ_b at the H···O bond critical point and the frequency of the N–H stretching vibrations ν(N–H)[a)] of heterodimers [2AmPyr+Mle] and [2AmNic+Mle] in water (PCM model). The H-bond enthalpy ΔH_{HB} (Eq. 1) and energy E_{HB} (Eq. 2) are given in the last two columns.

H-bond[a)]	R(H···O), Å	ν(N–H)[b)], cm^{-1}	ρ_b, a.u.	–ΔH_{HB}, kJ/mol	E_{HB}, kJ/mol
[2AmPyr+Mle]					
N1–H1···O1	1.777	2823 (2292)	0.0408	26.0	28.7
N2–H2···O2	1.859	3224[d)] (1360)	0.0324	22.7	24.3
[2AmNic+Mle]					
N1–H1···O1	1.718	2817 (2080)	0.0457	28.9	33.8
N2–H2···O2	1.870	3182[c)] (1103)	0.0324	22.3	23.5

[a)] scaling factor is 0.9648; [b)] see Fig. S4; [c)] IR intensity (km/mol) is given in parenthesis;

[d)] asymmetric stretching vibrations of the NH_2 group

Table S2. B3LYP/6-31G** values of the (H···O) distance R(H···O), the electron density ρ_b at the H···O bond critical point and the frequency of the N–H/O–H stretching vibrations ν(N–H)/ν(O–H)[a)] of trimers [2AmPyr+Mle+2AmPyr] and [2AmNic+Mle+2AmNic+H_2O] in water (PCM model). The H-bond enthalpy ΔH_{HB} (Eq. 1) and energy E_{HB} (Eq. 2) are given in the last two columns.

H-bond[b)]	R(H···O), Å	ν(N–H) /ν(O–H)[c)], cm^{-1}	ρ_b, a.u.	–ΔH_{HB}, kJ/mol	E_{HB}, kJ/mol
[2AmPyr+Mle+2AmPyr]					
N1–H1···O1	1.738	2837 (2488)	0.0538	27.9	42.6
N2–H2···O2	1.950	3270 (1132)	0.0378	19.6	30.5
O1 ··H3A–N2A	1.870	3230 (1382)	0.0341	22.3	27.0
[2AmNic+Mle+2AmNic+H_2O]					
N1–H1···O1	1.771	2902 (1868)	0.0636	26.3	49.8
N2–H2···O2	1.852	3217[d)] (1385)	0.0394	23.0	30.7
O2 ··H–Ow	1.710	3217[d)] (1385)	0.0399	29.3	33.2
Ow···H–OA	1.532	2526[e)] (1451)	0.0603	40.9	46.4

[a)] scaling factor is 0.9648; [b)] see Fig. S4; [c)] IR intensity (km/mol) is given in parenthesis;

[d)] asymmetric stretching vibrations of the NH_2 group strongly coupled with Ow–H stretch;

[e)] OA–H stretching vibration strongly coupled with intramolecular H-bond stretch

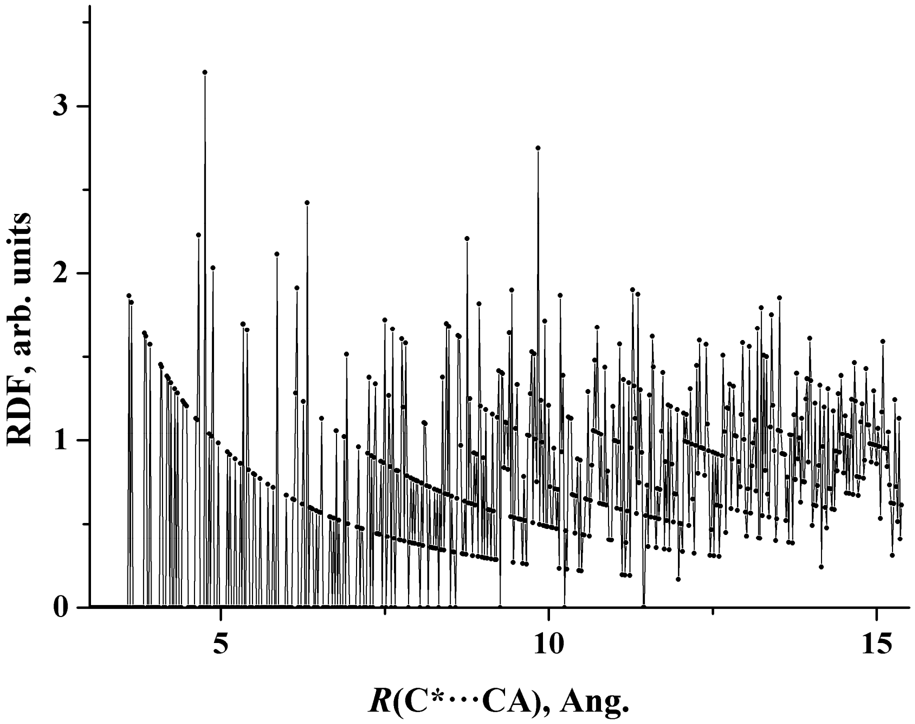

Figure S5. The radial distribution function of the *R*[C*···CA] distance obtained from the 100 ns *NPT* simulations of 2AmPyr and Mle in the 2x1x1 cell.

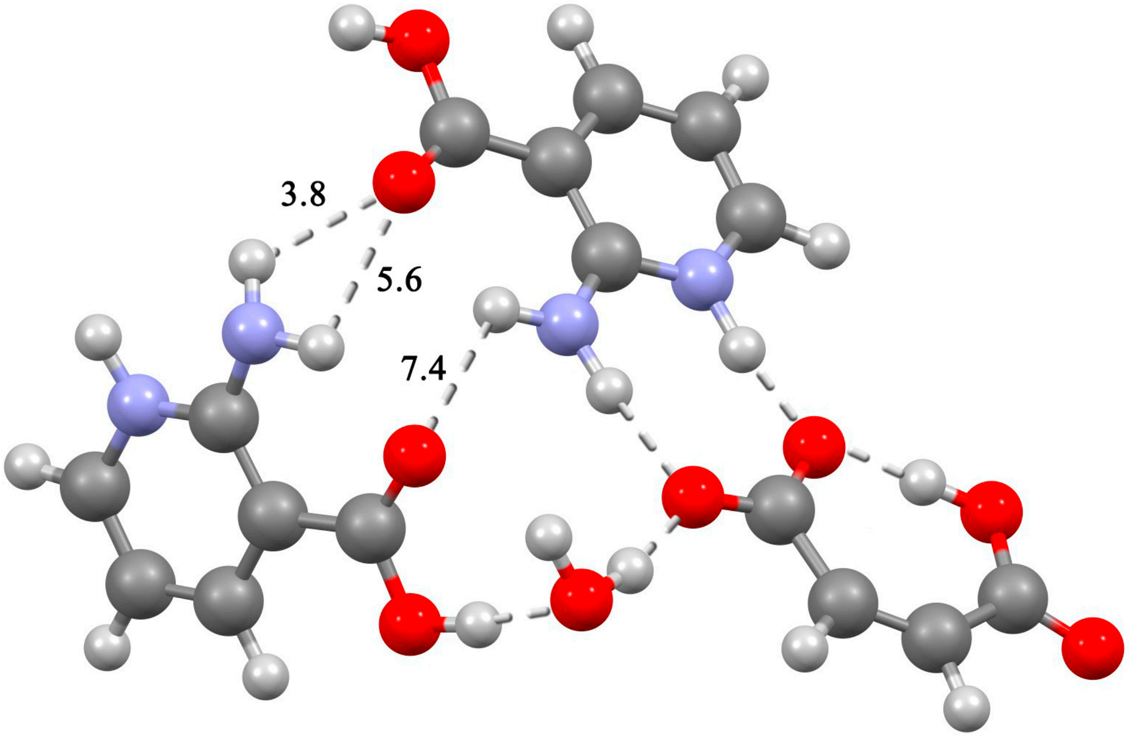

Figure S6. Several weak intermolecular interactions between two 2AmNic cations identified by the Bader electron density analysis followed by formula (2). Units are kJ/mol.

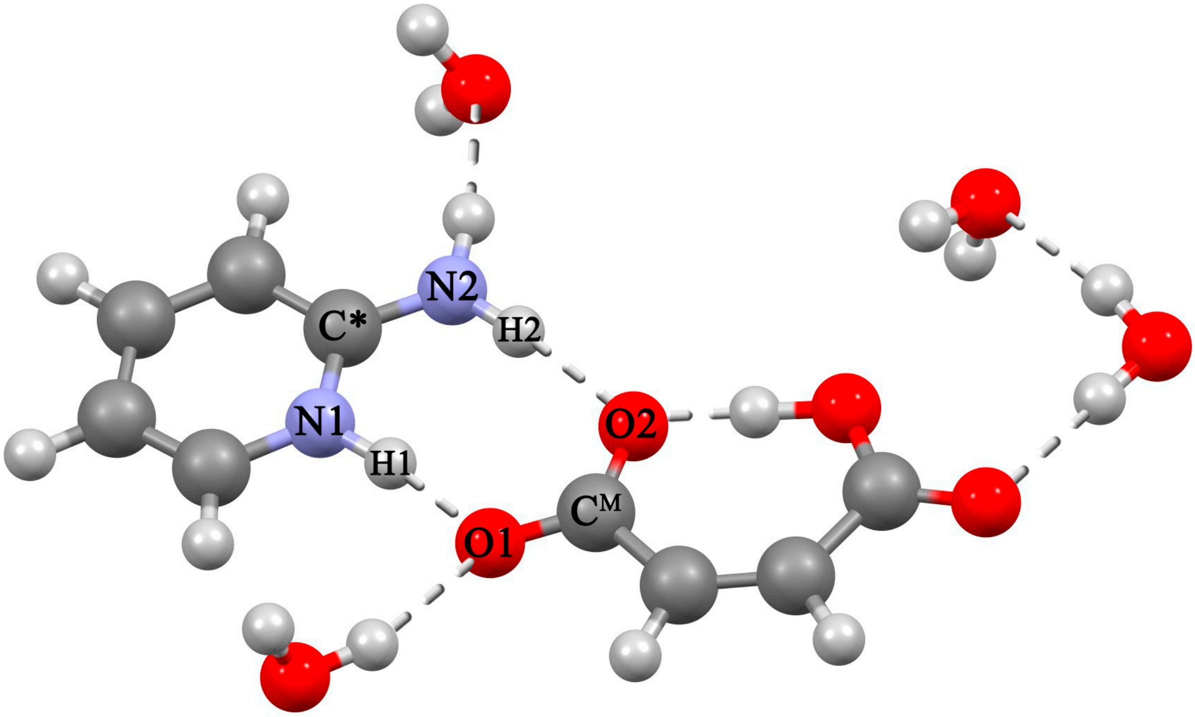

Figure S7. Structure of the [2AmNic+Mle] dimer hydrated with water molecules used in the calculation of the IR spectrum within the discrete-continuum approximation.

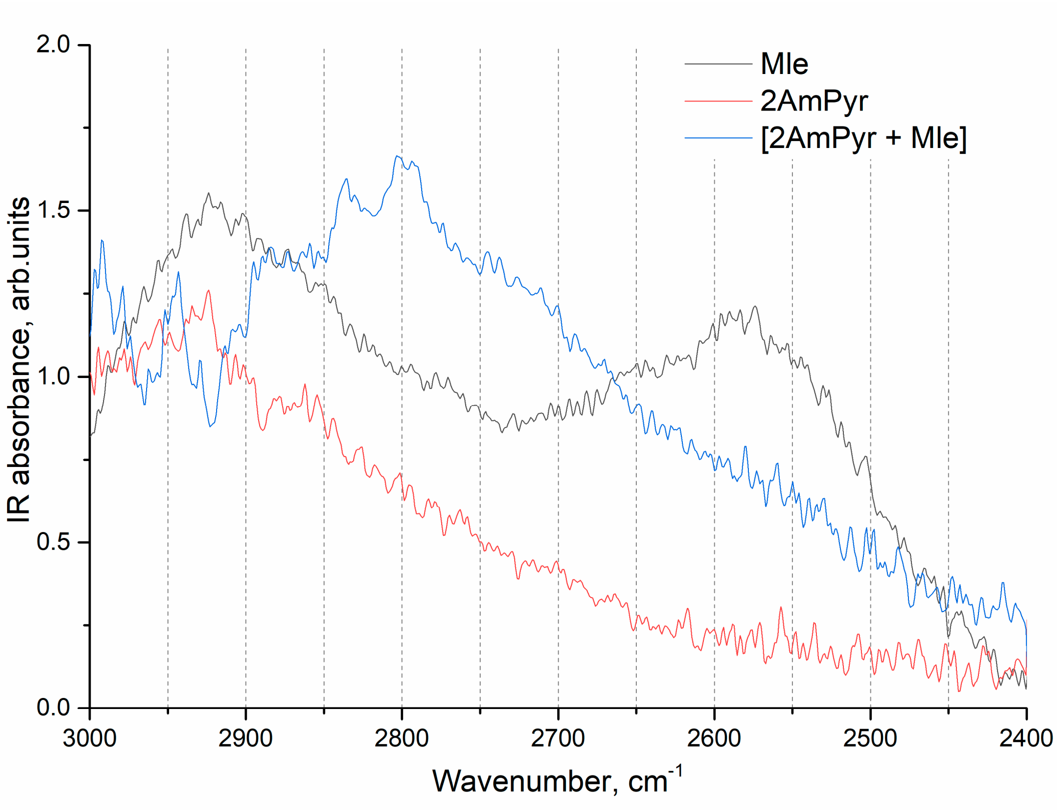

Figure S8. Comparison of the spectrum of [2AmPyr + Mle] with the spectrum of an aqueous solution of 2AmPyr and Mle of the same concentration in the range of 3000 – 2400 cm^{-1}.

References

s1. Shih, O.; England, A.H.; Dallinger, G.C.; Smith, J.W.; Duffey, K.C.; Cohen, R.C.; Prendergast, D.; Saykally, R.J. Cation-cation contact pairing in water: Guanidinium. *J. Chem. Phys.* **2013**, *139*, 035104.

s2. Rozanska, X.; Chipot, C. Modeling ion–ion interaction in proteins: A molecular dynamics free energy calculation of the guanidinium-acetate association. *J. Chem. Phys.* **2000**, *112*, 9691-9694.

s3. Debiec, K.T.; Gronenborn, A.M.; Chong, L.T. Evaluating the Strength of Salt Bridges: A Comparison of Current Biomolecular Force Fields. *J. Phys. Chem. B* **2014**, *118*, 6561−6569.

s4. Chowdhury, R.; Nandi, S.; Halder, R.; Jana, B.; Bhattacharyya, K. Structural relaxation of acridine orange dimer in bulk water and inside a single live lung cell. *J. Chem. Phys.* **2016**, *144*, 065101.

s5. Mak, C.H. Unraveling Base Stacking Driving Forces in DNA. *J. Phys. Chem. B* **2016**, *120*, 6010−6020.

s6. Kruchinin, S.E.; Fedotova, M.V. Ion Pairing of the Neurotransmitters Acetylcholine and Glutamate in Aqueous Solutions. *J. Phys. Chem. B* ***2021***, *125*, *11219*-11231.

Section S2. Topological file of the maleic acid monoanion.

```
[ moleculetype ]
; Name            nrexcl
maleate            3

[ atoms ]
;   nr      type  resnr residue  atom   cgnr     charge       mass
     1   opls_268      1    MAL    O1      1    -0.823123   15.9990
     2   opls_270      1    MAL    H1      1     0.614049    1.0080
     3   opls_269      1    MAL    O2      1    -0.707777   15.9990
     4   opls_267      1    MAL    C1      1     0.907584   12.0110
     5   opls_142      1    MAL    C2      1    -0.327958   12.0110
     6   opls_144      1    MAL    H2      1     0.144250    1.0080
     7   opls_142      1    MAL    C3      1    -0.327958   12.0110
     8   opls_144      1    MAL    H3      1     0.144250    1.0080
     9   opls_267      1    MAL    C4      1     0.907584   12.0110
    10   opls_268      1    MAL    O3      1    -0.823123   15.9990
    11   opls_269      1    MAL    O4      1    -0.707777   15.9990

; Stretchings
[ bonds ]
     1    2   1     0.1204       705.913
     1    4   1     0.1298    434106.036
     3    4   1     0.1235    622576.711
     4    5   1     0.1516    220874.352
     5    6   1     0.1097    322191.879
     5    7   1     0.1348    484343.743
     7    8   1     0.1097    322191.879
     7    9   1     0.1515    220874.352
     2   10   1     0.1201       705.913
     9   10   1     0.1298    434106.036
     9   11   1     0.1235    622576.711
```

```
; Bendings
[ angles ]
     2    1    4   1    111.60     301.9870
     1    2   10   1     178.64     184.6965
     1    4    3   1    124.84     867.1869
     1    4    5   1    118.23     409.6539
     3    4    5   1    116.93     300.2586
     4    5    6   1    111.17     330.6109
     4    5    7   1    130.85     623.4973
     6    5    7   1    117.98     305.0995
     5    7    8   1    117.99     305.0995
     5    7    9   1    130.85     623.4973
     8    7    9   1    111.16     330.6109
     7    9   10   1     118.24     409.6539
     7    9   11   1     116.96     300.2586
     2   10    9   1     111.58     301.9870
    10    9   11   1     124.80     867.1869

; Torsions
[ dihedrals ]
     2   1   4   3   1     179.97      13.451   2
     2   1   4   5   1     179.97       3.851   2
     4   1   2  10   1     179.21       0.011   2
     1   2  10   9   1     180.80       0.011   2
     1   4   5   6   1     180.04      -3.117   2
     1   4   5   7   1     180.04       4.013   2
     3   4   5   6   1     180.03      -0.345   2
     3   4   5   7   1     180.04      -7.520   2
     4   5   7   8   1     179.99      29.536   2
     4   5   7   9   1     180.00       3.652   2
     6   5   7   8   1     180.00      19.789   2
     6   5   7   9   1     180.01      29.536   2
     5   7   9  10   1     179.97       4.013   2
     5   7   9  11   1     179.97      -7.520   2
```

8 7 9 10 1 179.98 -3.117 2
8 7 9 11 1 179.98 -0.345 2
7 9 10 2 1 180.01 3.851 2
11 9 10 2 1 180.01 13.451 2
5 1 3 4 2 0.0 594.858
5 4 7 6 2 -0.0 124.419
9 5 8 7 2 -0.0 136.508
9 7 11 10 2 -0.0 873.561

; Nonbonded terms
[pairs]
; 1-4 interactions
1 6 2 0.833 0.000 0.000 0.0000 0.0000000
1 7 2 0.833 0.000 0.000 0.0000 0.0000000
1 9 2 0.833 0.000 0.000 0.0000 0.0000000
2 3 2 0.833 0.000 0.000 0.0000 0.0000000
2 5 2 0.833 0.000 0.000 0.0000 0.0000000
2 7 2 0.833 0.000 0.000 0.0000 0.0000000
2 11 2 0.833 0.000 0.000 0.0000 0.0000000
3 6 2 0.833 0.000 0.000 0.0000 0.0000000
3 7 2 0.833 0.000 0.000 0.0000 0.0000000
4 8 2 0.833 0.000 0.000 0.0000 0.0000000
4 9 2 0.833 0.000 0.000 0.0000 0.0000000
4 10 2 0.833 0.000 0.000 0.0000 0.0000000
5 10 2 0.833 0.000 0.000 0.0000 0.0000000
5 11 2 0.833 0.000 0.000 0.0000 0.0000000
6 8 2 0.833 0.000 0.000 0.0000 0.0000000
6 9 2 0.833 0.000 0.000 0.0000 0.0000000
8 10 2 0.833 0.000 0.000 0.0000 0.0000000
8 11 2 0.833 0.000 0.000 0.0000 0.0000000
1 8 2 1.000 0.000 0.000 0.0000 0.0000000
1 11 2 1.000 0.000 0.000 0.0000 0.0000000
2 6 2 1.000 0.000 0.000 0.0000 0.0000000
2 8 2 1.000 0.000 0.000 0.0000 0.0000000

```
  3    8   2    1.000     0.000     0.000      0.0000       0.0000000
  3    9   2    1.000     0.000     0.000      0.0000       0.0000000
  3   10   2    1.000     0.000     0.000      0.0000       0.0000000
  3   11   2    1.000     0.000     0.000      0.0000       0.0000000
  4   11   2    1.000     0.000     0.000      0.0000       0.0000000
  6   10   2    1.000     0.000     0.000      0.0000       0.0000000
  6   11   2    1.000     0.000     0.000      0.0000       0.0000000

; Exclusions from default nonbonded
[ exclusions ]
; ai       aj
    1    2
    1    3
    1    4
    1    5
    1    6
    1    7
    1    8
    1    9
    1   10
    1   11
    2    3
    2    4
    2    5
    2    6
    2    7
    2    8
    2    9
    2   10
    2   11
    3    4
    3    5
    3    6
    3    7
```

3	8
3	9
3	10
3	11
4	5
4	6
4	7
4	8
4	9
4	10
4	11
5	6
5	7
5	8
5	9
5	10
5	11
6	7
6	8
6	9
6	10
6	11
7	8
7	9
7	10
7	11
8	9
8	10
8	11
9	10
9	11
10	11
